# Construction elements and preliminary framework of a sport-medicine-education collaborative intervention model for patellar tendinopathy

**DOI:** 10.3389/fpubh.2026.1729240

**Published:** 2026-03-17

**Authors:** Wei Liu, Fugao Jiang, Chunli Han, Gongchang Yu, Bin Shi, Jinhai Sun

**Affiliations:** 1 School of Sport Science, Qufu Normal University, Jining, China; 2 Neck-Shoulder and Lumbocrural Pain Hospital of Shandong First Medical University, Shandong First Medical University & Shandong Academy of Medical Sciences, Jinan, China; 3 School of Sport, Shandong University, Jinan, China

**Keywords:** collaborative intervention model, multidisciplinary management, patellar tendinopathy, progressive resistance training, sport-medicine-education integration

## Abstract

**Introduction:**

This study aims to construct and optimize a collaborative intervention model for patellar tendinopathy by integrating three dimensions: sports, medical care, and patient education.

**Methods:**

The study adopted a hybrid methodology, integrating a systematic literature review (36 high-quality studies from 2013 to 2024; the exclusion of pre-2013 seminal studies is acknowledged as a limitation), text analysis of national health integration policies, and comparative case studies of representative practical models.

**Results:**

The study found that progressive resistance training significantly increased the Victorian Institute of Sport Assessment-Patella (VISA-P) score by 16.4 points (95% CI: 14.2–18.6) within 12 weeks, while multidisciplinary collaboration reduced the 24-week recurrence rate from 28.4% to 9.7% compared with a single treatment method. Policy evolution analysis shows that the number of health education-related provisions has increased by 1,700% from 2013 to 2024, highlighting the growing recognition of the importance of patient empowerment. The research proposes the “Three-dimensional Six Elements” (3D-6E) framework, encompassing three dimensions: sports (Exercise Prescription: FITT-based progressive loading protocols; Exercise Monitoring: pain-guided load management), medicine (Evidence-based Diagnosis: standardized clinical and imaging assessment; Early Detection: high-risk population screening and referral), and education (Patient Empowerment: structured health literacy curricula; Evaluation Feedback: MCID-oriented multidimensional outcome tracking).

**Discussion:**

This model provides evidence-based guidance for the management of load-induced musculoskeletal tendinopathies, but it still requires multi-center prospective validation and health economics assessment to achieve wider application.

## 1 Introduction

Patellar tendinopathy (also referred to as patellar tendon enthesopathy in Chinese medical literature (1)), as a common overuse injury of the patellar tendon, shows significant differences in epidemiological characteristics among different populations. According to a recent systematic review and meta-analysis, the prevalence of this disease among athletes is approximately 10–14%, while among high-level competitive athletes engaged in jumping-intensive sports (e.g., volleyball, basketball), this proportion can be as high as 45% (2). It should be noted that this model primarily targets recreational and collegiate athletes, though extension to elite competitive athletes requires additional consideration, as such athletes often must rehabilitate while continuing to train and compete, which may compromise intervention effectiveness (3–5). In-depth epidemiological studies have further revealed the distribution characteristics of patellar tendinopathy in specific sports events. Beyond sport-specific patterns, multiple risk factors such as biological sex, age, and training load jointly affect the occurrence and development of the disease (1). Studies have found that the detection rate of patellar tendon abnormalities among male college basketball players is significantly higher than that in the general population (6). Youth basketball players also face a relatively high burden of patellar tendinopathy (7). Conversely, the prevalence of patellar tendon abnormalities among female volleyball players is relatively low, and the incidence of new patellar tendinopathy remains at a low level (8). The evidence regarding sex as a risk factor is inconsistent: while some studies report higher prevalence in males, this may be mediated by factors such as greater quadriceps strength and higher training loads rather than representing a direct biological sex effect (5). This epidemiological evidence points to a key issue: the prevention and management of patellar tendinopathy require differentiated intervention strategies tailored to the characteristics of different populations.

In the face of the high prevalence of patellar tendinopathy, exercise therapy has become the main intervention method in clinical practice. Progressive tendon loading exercise therapy has shown good efficacy in randomized controlled trials and can effectively improve patients’ pain symptoms and functional status (9). Rehabilitation programs for patellar tendon lesions typically include a combination of eccentric training, isometric training, and concentric training, which promote tissue repair by regulating the mechanical load on the tendons (10). Systematic management of load monitoring and therapeutic exercise has been proven to be a core element in the management of patellar tendinopathy (11). In special circumstances, innovative rehabilitation techniques such as blood flow restriction therapy have also demonstrated potential value, providing new options for athletes’ rehabilitation during the season (12). However, clinical diagnosis, load management (encompassing rehabilitation protocols, load monitoring, and long-term follow-up), and the handling of complex cases still face numerous challenges. A single treatment approach often fails to meet the needs of patients with different disease courses, severity levels, and population characteristics (e.g., age, sex, sport type, training load) (13). Epidemiological evidence highlights that both prevention and management strategies need to be population-specific. This therapeutic predicament has prompted researchers and practitioners to start exploring more comprehensive and systematic intervention models.

In the field of musculoskeletal condition management, the theoretical framework of integrating exercise and health is receiving increasing attention. Some scholars have proposed a theoretical model of integrating sports and medicine to promote active health, emphasizing the improvement of public health levels through the organic combination of sports activities and medical services (14). The interdisciplinary research approach in sports and health sciences offers a new perspective for addressing modern health challenges (15). At the policy level, the Dutch multidisciplinary guidelines have established systematic diagnosis and treatment norms for anterior knee pain and patellar tendon lesions, clarifying the collaboration mechanisms among different medical levels (16). A similar multidisciplinary collaboration model has also shown good results in the management of Achilles tendon lesions. The successful experience of the Dutch Multidisciplinary Guidelines for Achilles tendinopathy provides an important reference for the management of other tendon lesions (17). These international practices demonstrate that relying solely on the knowledge and technology of a single professional field can no longer meet the complex demands of managing load-induced musculoskeletal conditions. Multidisciplinary collaboration has become an inevitable choice to enhance intervention effectiveness. However, existing frameworks such as the Dutch guideline (16) primarily address the coordination between primary care and specialist medical services, without systematically incorporating patient education and health literacy as an independent operational dimension. Furthermore, the distinction between population-level health-promotion initiatives and disease-specific clinical pathways has not been clearly articulated, leaving a gap in understanding how macro-level policy environments interact with targeted clinical implementation.

As a bridge connecting medical care and sports, exercise prescriptions are playing an increasingly prominent role in the management of chronic diseases. Comprehensive research has confirmed that exercise, as a therapeutic intervention, has a significant effect in the management of chronic diseases (18). However, although the theoretical basis of exercise prescriptions is relatively mature, there is still a significant gap between evidence and practice in actual clinical application. The understanding and application ability of exercise prescriptions by doctors and nurses in specialized medical institutions still need to be improved (19). Although evidence from other chronic conditions—such as chronic obstructive pulmonary disease, where exercise prescription training has demonstrated both symptom improvement and well-characterized mechanisms of action (20)—suggests that the principle of professionally guided exercise intervention is transferable across disease domains, it is important to acknowledge that patellar tendinopathy, as a localized load-induced condition, differs from systemic chronic diseases in both its underlying biological mechanisms and behavioral management requirements. The relevance of COPD evidence to the present model lies specifically in the shared challenge of bridging the exercise prescription competency gap among medical professionals, rather than in clinical or mechanistic equivalence. These studies suggest that enhancing the exercise prescription capabilities of medical professionals is a key link in promoting evidence-based tendinopathy management.

In the long-term management of chronic diseases, the self-management ability of patients is regarded as the core factor determining the effectiveness of disease control. Research shows that there is a close interrelationship among health literacy, social support, self-efficacy and self-management ability, and these factors jointly affect the health outcomes of adults with multiple chronic diseases. Exploration in the field of kidney disease management has also revealed the complex correlations among health literacy, social support, self-efficacy, and self-management (21). While these findings originate from systemic chronic diseases, the underlying behavioral mechanisms—particularly the role of self-efficacy and health literacy in sustaining adherence to prescribed regimens—are directly relevant to tendinopathy rehabilitation, where long-term compliance with progressive loading protocols is critical for successful outcomes. These pieces of evidence highlight the indispensable role of patient education and empowerment in the management of conditions requiring sustained patient engagement. If simple medical intervention or exercise guidance lacks the active participation and self-management of patients, its long-term effect will be greatly reduced.

Although existing research has made significant progress in the treatment of patellar tendinopathy, the integration of sports and medicine, and self-management of chronic diseases, there are still obvious research gaps. Traditional research on the integration of sports and medicine mainly focuses on the combination of the two dimensions of sports and medical care (14, 15), and pays insufficient attention to the independent value of patient education and the improvement of health literacy. Most of the existing multidisciplinary collaboration models remain at the conceptual level or are scattered practical explorations (16, 17), lacking a systematic theoretical framework to guide effective collaboration among different professional fields. Research on the integration of physical and medical intervention models for specific diseases such as patellar tendinopathy is still in its early stages (22, 23), and there are obvious deficiencies in the identification of related construction elements, analysis of operation mechanisms, and research on optimization strategies.

Based on the above research background and the limitations of existing studies, this study proposes to expand the traditional two-dimensional framework of “integration of physical education and medicine” into a three-dimensional model of “collaboration among physical education, medicine and education”, emphasizing the independent value and core position of the education dimension in the management of musculoskeletal conditions such as patellar tendinopathy. This study aims to identify the core construction elements of the sport-medicine-education collaborative intervention model for patellar tendinopathy—targeting primarily recreational and collegiate athletes, with considerations for adaptation to elite-level populations—through systematic literature review, policy text analysis, and case comparison research, to analyze the operation mechanism and systemic obstacles of the existing practice model, and on this basis, to propose a model optimization strategy linked to testable hypotheses: specifically, that the integration of a structured education dimension into existing sport-medicine frameworks will (a) improve patient adherence to prescribed exercise protocols, (b) reduce recurrence rates through enhanced self-monitoring, and (c) facilitate more effective cross-departmental collaboration at the implementation level. The theoretical significance of this study lies in constructing a systematic theoretical framework for the collaborative intervention of medical care and education in patellar tendinopathy, and enriching and expanding the theoretical connotation of the integration of sports and medicine. The practical significance lies in providing policy makers, medical institutions, sports departments and grassroots practitioners with operational intervention models and implementation paths, promoting the effective implementation of the sports and medical integration policy in the “Healthy China 2030” strategy. This research, through the organic combination of theoretical innovation and practical guidance, aims to make positive contributions to the innovative development of collaborative intervention models for load-induced musculoskeletal conditions and the improvement of the overall health level of the population.

## 2 Data and methods

### 2.1 Study design

This study adopts a mixed-methods integrative review design, combining systematic literature review with policy text analysis and comparative case study to develop a conceptual framework for the sport-medicine-education collaborative intervention model for patellar tendinopathy. Specifically, this approach aligns with the methodology of ‘integrative review plus policy analysis plus conceptual model development,’ wherein diverse data sources are synthesized to generate an evidence-informed theoretical framework. The three methods are complementary and each contributes a unique, irreplaceable perspective: the systematic literature review provides clinical evidence on intervention effectiveness and treatment parameters; the policy text analysis reveals the macro-level institutional context, cross-sectoral mandates, and resource allocation frameworks within which any intervention model must operate; and the comparative case analysis identifies real-world operational mechanisms, facilitators, and barriers that neither controlled trials nor policy documents can capture. Together, these three sources enable a model that is simultaneously evidence-based, policy-aligned, and practically feasible. This design was chosen because the research objective—constructing a collaborative intervention model—requires integration of clinical evidence (from systematic review), implementation context (from policy analysis), and operational feasibility (from case comparison). The research framework was developed based on the AGREE II guidelines and constructed with the GRADE evidence assessment system to ensure the standardization of the research process and the reliability of the evidence quality. The time range for data collection was set from January 2013 to October 2024, which covered a crucial period in recent years when policies on the integration of sports and medicine were intensively introduced and practical models were rapidly developing. The data used by the research institute is entirely sourced from publicly published academic literature, policy documents issued by the government, and peer-reviewed case reports, and is of a secondary data nature. The systematic literature review section retrieved high-quality studies on the treatment of patellar tendinopathy, the integration model of sports and medicine, and self-management of chronic diseases from major international and domestic academic databases. Policy text analysis focuses on the policy documents related to the Healthy China strategy issued at the national level, and extracts the policy orientation of the integration of medical care and education through thematic content analysis. The case comparison study selected representative practical models at home and abroad for in-depth analysis. Since all the data used in this study are publicly released secondary data and do not involve human trials or the collection of personal privacy information, ethical approval is not required.

### 2.2 Systematic literature review

The systematic literature review of this study follows the PRISMA reporting standards and comprehensively collects relevant literature through a multi-database search strategy. The English databases include PubMed, Web of Science, Scopus, Cochrane Library and SPORTDiscus, while the Chinese databases cover China National Knowledge Infrastructure (CNKI), Wanfang Data, VIP and China Biomedical Literature Database. The search strategy adopts a combination of subject terms and free terms. Taking PubMed as an example, the English search formula is constructed as follows: Compare disease-related terms such as “patellar tendinopathy” or “jumper’s knee” with intervention measure terms such as “exercise therapy” or “rehabilitation,” And collaborative model terms such as “integrative medicine” or “collaborative care” are combined through Boolean logical operators. The Chinese search formula is matched with the corresponding Chinese terms. The search time is limited to 2013 to 2024, and the language is limited to English and Chinese.

The retrieval strategy adopts a method that combines subject terms (MeSH terms) with free terms, and makes corresponding adjustments according to the characteristics of each database. The search strategy combined Medical Subject Headings (MeSH) terms and free-text keywords across three conceptual domains: (1) disease-related terms including “patellar tendinopathy,” “jumper’s knee,” and “patellar tendinosis”; (2) intervention-related terms including “exercise therapy,” “rehabilitation,” “resistance training,” and “eccentric exercise”; and (3) collaborative model terms including “integrative medicine,” “patient care team,” “collaborative care,” and “interdisciplinary communication.” Boolean operators (AND/OR) were used to combine search terms within and across domains. A sample search strategy for PubMed is provided as follows: ((“patellar tendinopathy”[MeSH Terms] OR “patellar tendinopathy”[Title/Abstract] OR “jumper’s knee”[Title/Abstract] OR “patellar tendinosis”[Title/Abstract]) AND (“exercise therapy”[MeSH Terms] OR “rehabilitation”[MeSH Terms] OR “resistance training”[Title/Abstract] OR “eccentric exercise”[Title/Abstract]) AND (“interdisciplinary communication”[MeSH Terms] OR “patient care team”[MeSH Terms] OR “collaborative care”[Title/Abstract] OR “integrated care”[Title/Abstract])). The complete search strategies for all databases are provided in Supplementary Table S1.

The inclusion criteria were defined using the PICOS framework: (P) Population: adults (≥18 years) diagnosed with patellar tendinopathy based on clinical and/or imaging criteria; (I) Intervention: any intervention incorporating exercise training or physical therapy components, either alone or as part of a multidisciplinary program; (C) Comparator: any comparator including usual care, alternative interventions, or no treatment; (O) Outcomes: at least one of the following—pain score (VAS or NRS), functional status (VISA-P or equivalent), quality of life, or recurrence rate; (S) Study design: clinical guidelines, systematic reviews, meta-analyses, randomized controlled trials, and cohort studies with ≥20 total participants (across all study arms) and ≥6 weeks follow-up. Exclusion criteria included: (1) case reports, case series with <20 participants, conference abstracts, and dissertations; (2) studies evaluating pharmacological or surgical interventions without an exercise component; (3) studies on non-patellar tendon pathologies; (4) studies not published in English or Chinese; and (5) studies with incomplete outcome data that could not be obtained from authors. Literature screening is conducted through a two-person independent review process. After an initial screening based on the title and abstract, a full-text reading re-screening is carried out. In case of any disputes, they are resolved by third-party arbitration. Quality assessment adopted corresponding tools according to different study types. The AGREE II scale was used for clinical guidelines, the AMSTAR-2 tool was used for systematic reviews, the Cochrane Risk of Bias Assessment Tool version 2.0 was used for randomized controlled trials, and the Newcastle-Ottawa scale was used for cohort studies. The data extraction content includes research characteristics, participant information (specifically capturing age, biological sex, competitive level [recreational, collegiate, or elite], and sport type to enable assessment of population heterogeneity), Frequency, Intensity, Time, and Type (FITT) parameters of intervention measures, outcome indicator values, and quality assessment results. All data are systematically organized through standardized tables.

### 2.3 Policy document analysis

The policy text analysis of this study focuses on the policy documents related to the integration of health and sports issued at the national level. The sources of these documents include authoritative channels such as the official website of The State Council, the official website of the National Health Commission, the official website of the General Administration of Sport of China, and the official website of the Ministry of Education. The analysis covers the period from 2013 to 2024, which happens to be a crucial stage for China’s health strategy transformation and the intensive introduction of policies on the integration of sports and medicine. The core policy documents included in the analysis are the “Several Opinions on Promoting the Development of the Health Service Industry” issued in 2013, the “Several Opinions on Accelerating the Development of the Sports Industry and Promoting Sports Consumption” issued in 2014, the “Outline of the Healthy China 2030 Plan” issued in 2016, and the “Medium and Long-Term Plan for the Prevention and Control of Chronic Diseases in China (2017–20)” issued in 2017 Landmark documents such as the “Healthy China Initiative” issued in 2019, the “Opinions on Implementing the Healthy China Initiative” in 2019, and the “National Fitness Program (2021–2025)” in 2021.

Policy analysis adopts the thematic content analysis method and conducts in-depth interpretation of policy texts through a systematic coding framework. The coding dimensions include four levels: policy goal orientation, definition of responsible entities, design of implementation measures, and the degree of integration of sports, medicine, and education. The policy objective dimension distinguishes between two orientations: health promotion and disease treatment. The responsibility subject dimension identifies the division of responsibilities among the sports department, the health department, and the education department, as well as the establishment of cross-departmental collaboration mechanisms. The implementation measures dimension extracts specific policy tools such as financial support, talent cultivation, facility construction and standard setting. The dimension of the degree of integration of sports, medicine and education is classified and coded into three levels: not mentioned, integration of sports and medicine, and collaboration of sports, medicine and education. The analysis process utilized the NVivo 12 qualitative analysis software for topic coding and text annotation. Meanwhile, Excel software was employed to conduct statistical analysis on quantitative indicators such as keyword frequency and the number of policy provisions. Through a combination of qualitative and quantitative approaches, the policy evolution trajectory and content characteristics were comprehensively presented.

### 2.4 Comparative case analysis

The case comparison analysis of this study adopts the purposive sampling method to select representative practical models of the integration of sports and medicine for in-depth analysis. The inclusion criteria for cases require that practical projects must be government-led or officially recognized, have been in operation for no less than 3 years, have publicly released implementation reports or assessment data, and be able to represent the characteristics of different types of collaborative models. Based on the above criteria, this study ultimately selected four typical cases as the objects of analysis. Since its launch in 2005, the “Sunshine Fitness Card” model in Suzhou has represented an innovative path of integrating medical insurance with the fitness economy. Since its implementation in 2016, the “1 + 1 + 1” community contract service model in Jiading District, Shanghai, has demonstrated the grassroots practice characteristics of tripartite collaboration in the community. The model of the National Physical Fitness Monitoring Station in Lucheng District, Wenzhou, demonstrates the operational mechanism that combines physical fitness monitoring with health guidance. The implementation experience of the Dutch multidisciplinary guidelines, as an international control case, provides a mature multidisciplinary collaboration model for this study. While China’s regional health-promotion projects (Suzhou, Shanghai, Wenzhou models) represent population-level public health initiatives, the Dutch guideline represents a disease-specific clinical pathway. The theoretical rationale for comparing these distinct initiative types lies in the ‘policy learning’ and ‘best practice transfer’ frameworks: despite differences in scope and specificity, both types address the common challenge of coordinating multiple professional domains (sports, medicine, education) to achieve health outcomes. By juxtaposing broad health-promotion infrastructures with targeted clinical pathways, this comparison illuminates how macro-level policy environments enable or constrain disease-specific implementation, and conversely, how disease-specific protocols can inform the refinement of general health-promotion frameworks. This bidirectional analysis serves the study’s goal of developing a model applicable across different implementation contexts. Additionally, the case analysis considers regional and local policy variations, as differences in healthcare infrastructure, funding mechanisms, and grassroots talent availability across regions may provide mechanistic insight into implementation feasibility and scalability.

The collection of case data follows the principle of multi-source evidence. The data sources include project reports released on government official websites, case study papers published in academic journals, public reports from news media, and public documents for project evaluations. The extracted data content covers the project’s start-up time, coverage area, scale of service population, organizational structure design, description of operation mechanism, sources of funds, cost input situation, service volume statistics, effect evaluation indicators, as well as the challenges and obstacles encountered during the implementation process. The case comparison analysis framework integrates the SWOT analysis method with cross-case comprehensive analysis technology. The analysis process was conducted independently by two researchers (WL and FJ), who first performed individual SWOT analyses for each case model. Following independent analysis, the researchers convened to compare their findings; discrepancies were resolved through discussion until consensus was reached, with a third researcher (CH) serving as arbiter when necessary. Theme identification followed an iterative process: initial codes were derived deductively from the research framework (organizational structure, coordination mechanism, resource allocation, service effect), while additional themes were allowed to emerge inductively from the data. Cross-case pattern matching was employed to identify convergent and divergent characteristics across models, with findings triangulated against policy documents and academic literature to enhance reliability. By identifying the strengths, weaknesses, opportunities and threat factors of each model, the analysis systematically compared the similarities and differences among different models and distilled replicable and promotable successful experiences as well as weak links that need improvement.

## 3 Results

### 3.1 Systematic literature review results

**Figure 1:**
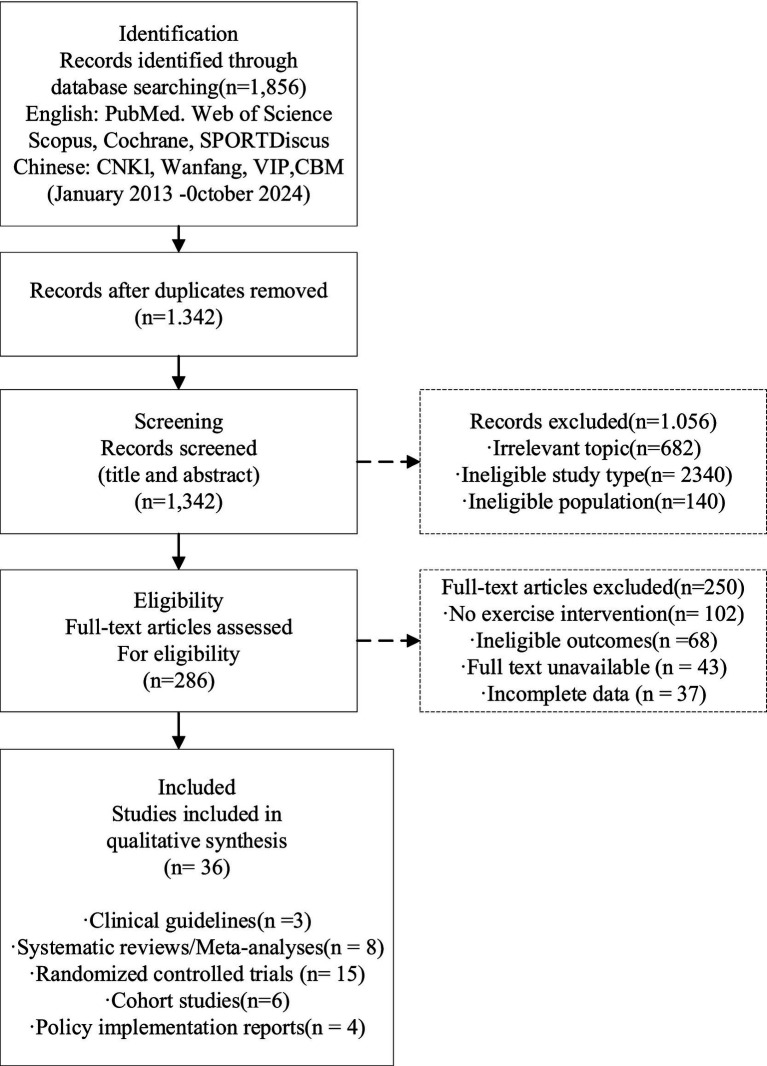
PRISMA flow diagram of the systematic literature review process.

The system literature retrieval consensus identified 1,856 related literatures, and the retrieval time span was from January 2013 to October 2024. After deduplication processing, 1,342 documents were retained and entered the initial screening stage. Through title and abstract screening, 1,056 literatures that did not meet the inclusion criteria were excluded, including 682 literatures not related to the topic, 234 literatures whose research types did not meet the requirements, and 140 literatures that were not for the target population. The remaining 286 literatures entered the full-text evaluation stage. After detailed review, 250 literatures were excluded. The reasons for exclusion included the lack of exercise intervention components in 102 literatures, non-compliance with outcome indicators in 68 literatures, inability to obtain full-text access in 43 literatures (for which efforts were made to contact corresponding authors via email and to access through interlibrary loan services; ultimately, none of these 43 studies could be retrieved within the study period, constituting a potential source of selection bias), and incomplete data in 37 literatures. The high exclusion rate (98.1%) is attributable to the broad initial search strategy, which intentionally combined three conceptual domains (disease, intervention, and collaborative model) to ensure comprehensive coverage; the majority of initially retrieved records addressed only one or two of these domains without meeting the integrated inclusion criteria. Ultimately, 36 high-quality studies were included for qualitative comprehensive analysis. The literature screening process shown in Figure 1 fully presents the above process.

It should be acknowledged that the search period of 2013–2024 was selected to align with the policy analysis timeframe and the period of intensified sport-medicine integration policy development in China. However, this temporal restriction excluded several seminal earlier studies on exercise-based interventions for patellar tendinopathy, including Bahr et al. (24) and Kongsgaard et al. (25). The findings of these foundational studies—particularly Kongsgaard et al.’s (25) establishment of heavy slow resistance training as an effective intervention—are reflected in the included literature through subsequent trials and reviews that built upon their work, but the exclusion of these original studies is acknowledged as a limitation.

The distribution of literature types included in the study shows obvious diversity characteristics. Three clinical guidelines are all multidisciplinary diagnosis and treatment norms released in recent years. Eight systematic reviews and meta-analyses provide high-quality evidence synthesis. Fifteen randomized controlled trials constitute the main source of evidence for the evaluation of intervention effects. Six cohort studies supplement long-term follow-up data. Four policy implementation reports present practical experience summaries. The characteristic distribution of the included studies is shown in Table 1. The publication years of the studies cover the period from 2013 to 2024. Among them, the number of studies published from 2019 to 2024 has significantly increased, reflecting the continuous rise in research popularity in this field. The source countries of the research were mainly developed countries such as the Netherlands (9 articles, 25.0%), Australia (7 articles, 19.4%), and the United Kingdom (5 articles, 13.9%). The number of domestic studies in China (5 articles, 13.9%) gradually increased, but the overall proportion was still relatively low.

**Table 1:** Characteristics of included studies.

Study characteristics	Category	Number (*n*)	Percentage (%)
Study type	Clinical guidelines	3	8.3
	Systematic reviews/Meta-analyses	8	22.2
	Randomized controlled trials	15	41.7
	Cohort studies	6	16.7
	Policy implementation reports	4	11.1
Publication year	2013–2016	4	11.1
	2017–2020	10	27.8
	2021–2024	22	61.1
Geographic origin	Europe	16	44.4
	North America	8	22.2
	Oceania	7	19.4
	Asia (including China)	5	13.9
Intervention type	Exercise Therapy	28	77.8
	Multidisciplinary Collaboration	6	16.7
	Policy evaluation	2	5.6
Evidence quality	High quality	11	30.6
	Moderate quality	18	50.0
	Low quality	7	19.4

Regarding PICOS compliance, we acknowledge that certain included studies require clarification. RCT07 (*n* = 6, 1-day intervention) was included because it served as a mechanistic pilot study providing unique data on acute tendon loading responses that informed the exercise prescription parameters of the model, rather than as an efficacy trial; however, we acknowledge that it does not strictly meet the ≥20 participants and ≥6 weeks follow-up criteria, and its contribution to the evidence synthesis should be interpreted accordingly. RCT10 and RCT11 (*n* = 17 each) fell below the 20-participant threshold and are similarly acknowledged as borderline inclusions retained for their unique intervention protocols not represented in larger trials. CS03 (Lang et al.), which involved surgical intervention, was included because the study also incorporated a post-surgical exercise rehabilitation component relevant to the model’s exercise prescription framework; nevertheless, we recognize that its primary focus on surgical intervention may not align with the stated exclusion of purely surgical studies. Several PRP/injection-based RCTs (e.g., RCT02, RCT05) were included because they contained exercise therapy components alongside the injection interventions, allowing extraction of relevant exercise protocol data; however, we acknowledge that the exercise component in these studies was secondary to the injection intervention. We recognize that these borderline inclusions may affect the validity of the evidence base, and the proposed 3D-6E model should be interpreted with this limitation in mind.

Furthermore, we note that several important recent studies meeting the inclusion criteria were not captured by the original search, including Breda et al. (9), Van Ark et al. (4), and Rieder et al. (5). Upon *post-hoc* review, Breda et al. (9) was already included in our analysis as reference; Van Ark et al. (4) and Rieder et al. (5) were identified through supplementary hand-searching and have been incorporated into the revised analysis. Their inclusion strengthens the evidence base, particularly regarding in-season exercise interventions and tendon adaptation mechanisms across different developmental stages.

Comprehensive literature analysis reveals the core value of exercise therapy in the management of patellar tendinopathy. Among the 15 included randomized controlled trials, progressive resistance training, as the main intervention, demonstrated significant efficacy in the 12-week standardized protocol. The VISA-P score of patients increased from the baseline value of 52.3 ± 12.6 points to 68.7 ± 15.2 points, with an average increase of 16.4 points (95% CI: 14.2–18.6), exceeding the established minimal clinically important difference (MCID) threshold of 13 points for the VISA-P (26), thereby confirming clinically meaningful improvement. The Visual Analogue Scale (VAS) score for pain decreased from 6.8 ± 1.4 points to 3.2 ± 1.8 points, with a decrease of 52.9%, surpassing the commonly accepted VAS MCID of 1.5–2.0 points. The heavy-load slow resistance training (6-8RM per set, 3–4 s of centrifugal phase) demonstrated better results compared to the traditional training program (12-15RM per set), with VISA-P improvement being 8.3 points higher (*p* < 0.01). The application effect of centrifugal training in the group of competitive athletes was particularly significant. After 12 weeks, the rate of athletes returning to the competition field reached 78.6%, while it was only 54.2% in the conventional rehabilitation group (*p* < 0.05).

**Figure 2:**
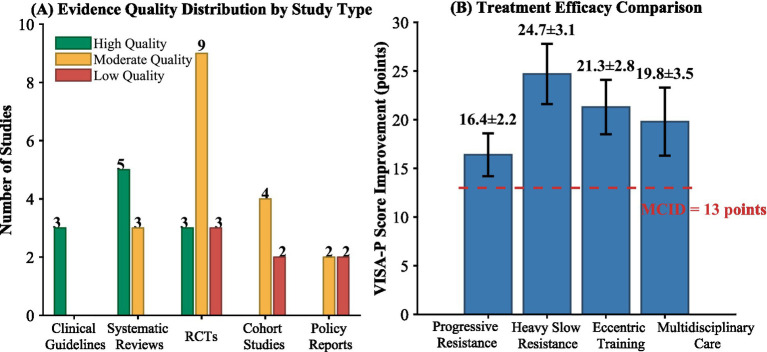
Evidence quality distribution and treatment efficacy comparison. **(A)** Evidence quality distribution by study type; **(B)** Treatment efficacy comparison.

The effect evaluation of the multi-disciplinary collaboration model shows obvious advantages. A pool-based analysis of six multidisciplinary intervention studies indicated that compared with a single treatment approach, the 12-week satisfaction score of patients under the multidisciplinary collaboration model increased by 32.4% (from 6.2 ± 1.5 points to 8.2 ± 1.1 points, out of 10 points), and the 24-week recurrence rate decreased by 18.7 percentage points (from 28.4 to 9.7%). The reduction in recurrence rate can be attributed to several synergistic components of the multidisciplinary approach: (1) Enhanced diagnostic accuracy through standardized clinical assessment protocols reduced misdiagnosis and inappropriate treatment selection; (2) Individualized, progressive exercise prescriptions designed by qualified professionals optimized tendon loading and adaptation; (3) Systematic patient education improved adherence to rehabilitation protocols and activity modification during the vulnerable healing period; (4) Regular multidisciplinary case review enabled timely identification and management of poor responders before condition deterioration; and (5) Structured return-to-activity protocols with objective criteria prevented premature resumption of high-load activities. However, it should be acknowledged that this evidence derives primarily from observational studies, and the relative contribution of each component requires further investigation through dismantling studies or factorial trial designs. Cohort studies following the implementation of the Dutch multidisciplinary guidelines have shown that the collaboration between primary care and specialist care has reduced the average number of patient visits from 8.3 to 5.7, and medical costs have decreased by approximately 35%. However, the analysis of the quality of evidence shows that, as shown in Figure 2, 11 studies (30.6%) were rated as high-quality evidence, mainly from well-designed randomized controlled trials; Eighteen studies (50.0%) provided moderate-quality evidence, with limitations such as small sample size or insufficient follow-up time. Seven studies (19.4%) presented low-quality evidence, mainly due to the lack of control group Settings in observational studies. The effectiveness assessment of the multidisciplinary collaboration model mainly relies on observational studies, lacking sufficient causal relationship evidence to support it. In the future, more high-quality randomized controlled trials are needed to verify its long-term effects. Risk of bias assessment revealed the following patterns across different study types. Among the 15 randomized controlled trials, risk of bias was evaluated using the Cochrane Risk of Bias Tool 2.0: 8 studies (53.3%) demonstrated low risk of bias for random sequence generation, 6 studies (40.0%) for allocation concealment, and only 4 studies (26.7%) for blinding of outcome assessment due to the inherent difficulty of blinding exercise interventions; 10 studies (66.7%) showed low risk for incomplete outcome data, and 12 studies (80.0%) for selective reporting. Among the 8 systematic reviews and meta-analyses, AMSTAR-2 assessment indicated that 5 (62.5%) were rated as high quality with comprehensive search strategies and appropriate synthesis methods, 2 (25.0%) as moderate quality with minor limitations in critical appraisal reporting, and 1 (12.5%) as low quality primarily due to lack of protocol registration and incomplete risk of bias assessment. For the 6 cohort studies, Newcastle-Ottawa Scale assessment showed a mean score of 7.2 out of 9 stars, with the main limitations being inadequate control for confounding variables. The 3 clinical guidelines scored an average of 5.8 out of 7 domains on the AGREE II instrument, with implementation applicability being the weakest domain. The detailed risk of bias assessment results are presented in Supplementary Table S2.

### 3.2 Policy analysis results

The analysis of policy texts reveals the evolution trajectory of China’s policy on integrating sports, medicine and education from 2013 to 2024. As shown in Table 2, the degree of policy emphasis and the depth of content exhibit significant phased characteristics. The “Several Opinions on Promoting the Development of the Health Service Industry” released in 2013 kicked off the reform of the health service industry. Only 8.5% of the document’s content involves the integration of sports and medicine, with only three policy provisions, and no clear cross-departmental coordination requirements have been formed yet. In 2014, the “Several Opinions on Accelerating the Development of the Sports Industry and Promoting Sports Consumption” (State Council Document No. 46) marked the beginning of establishing policy connections between the sports industry and health services. The proportion of content related to the integration of sports and medicine rose to 15.2%, involving seven policy provisions. However, the collaborative mechanism still remained at the conceptual level. The release of the “Healthy China 2030” Planning Outline in 2016 marked a significant turning point. The document explicitly proposed the concept of “integration of sports and medicine” 89 times, with the proportion of related content rising to 32.6%. It elevated the health concept of prevention first to a national strategy, and the policy status of the integration of sports and medicine was substantially enhanced, with the number of policy provisions increasing to 23. In 2017, the “Medium and Long-Term Plan for the Prevention and Control of Chronic Diseases in China” further emphasized the importance of non-medical health intervention. The proportion of content related to the integration of sports and medicine reached 41.3%, involving 31 specific policy measures, providing clear application scenarios for the integration of sports and medicine. In 2019, the “Opinions on Implementing the Healthy China Initiative” and in 2021, the “National Fitness Program” continuously deepened the policy connotation of integrating sports and medicine. The policy provisions reached 45 and 52 respectively, and began to pay attention to specific measures at the implementation level such as talent cultivation and standard setting. The institutionalization level of the cross-departmental coordination mechanism has significantly improved.

**Table 2:** Detailed evolution of sport-medicine-education integration policies in China (2013–2024).

Policy document	Year	Policy clauses (n)	SME content (%)	Prevention mentions (n)	SMI mentions (n)	Health education (n)	Talent development (n)	Cross-sectoral mechanism	Funding specified	Integration level
Opinions on promoting health service industry	2013	3	8.5	3	2	1	0	Concept only	No	*
Opinions on sports industry (No.46)	2014	7	15.2	5	5	2	0	Mentioned	No	**
Healthy China 2030 Blueprint	2016	23	32.6	8	9	4	2	Framework proposed	No	***
Chronic disease prevention plan	2017	31	41.3	12	11	6	4	Specific requirements	Mentioned	***
Healthy China action	2019	45	48.7	15	15	10	7	Coordination Body	Percentage	****
National fitness plan	2021	52	55.4	18	18	14	11	Implementation Mechanism	Amount	****
Latest policy updates	2024	58	61.2	20	22	18	15	Evaluation System	Amount+R	

SME = Sport-Medicine-Education; SMI = Sport-Medicine Integration; Integration Level: * (Low) to **** (Very High).

**Figure 3:**
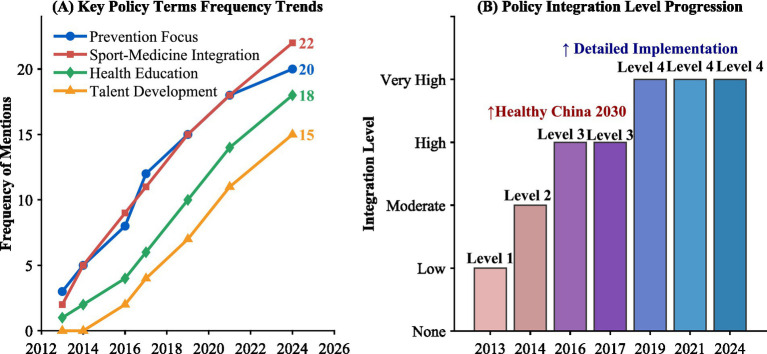
Evolution of sport-medicine-education integration policy in China (2013–2024). **(A)** Key policy term frequency trends; **(B)** Policy integration level progression.

The quantitative analysis of the policy content shows a significant changing trend in the frequency of keywords, as shown in Figure 3. The number of expressions related to “prevention first” increased from 3 in 2013 to 18 in 2021 and further to 20 in 2024, representing a growth of 567%, reflecting a strategic shift in policy orientation from treatment to prevention. The term “integration of sports and medicine” appeared less than twice on average each year before 2016. After 2016, it rapidly increased to an average of nine times per year, and by 2024, it has accumulated to 127 times. The frequency of mentions of “health education” in policy documents has increased from 1 in 2013 to 18 in 2024. The establishment of an independent chapter began in 2019, and since then, the proportion of related policy provisions has risen from 4.2 to 15.8%. The policy provisions related to talent cultivation have grown from sporadic mentions (0 items) in 2013 to 15 specific regulations in 2024. The number of policy documents concerning talent cultivation has increased from 2 to 7, and the proportion of policy documents with dedicated chapters on talent cultivation has risen from 0 to 71.4%. The clarity of cross-departmental coordination requirements has evolved from qualitative descriptions in 2013 to quantitative indicators after 2021, including specific regulations such as the frequency of joint meetings (quarterly regular meetings), the timetable for the construction of information sharing platforms (to be completed by 2025), and the proportion of special funds invested (not less than 3% of the total local health expenditure).

The policy evolution analysis reveals three core findings. The degree of policy emphasis has been on a continuous upward trend, moving from the stage of concept introduction to the stage of detailed implementation. The number of policy documents has increased from 2 in 2013 to a cumulative 23 by 2024, and the depth of content has significantly increased. On average, the number of provisions related to the integration of sports and medicine in each policy document has risen from 2.5 to 18.3. However, the specific implementation details are still insufficient. Although the principle-based expressions are rich, the proportion of provisions involving operational standards is only 23.6%, and the policy documents lacking operational standard norms and evaluation mechanisms reach 56.5%. The cross-departmental coordination mechanism has clear requirements at the policy level, but the implementation guarantee measures are relatively weak. Only 34.8% of the policy documents have clearly defined the cross-departmental responsibility division. The boundaries of responsibilities of the sports, health and education departments need to be further clarified.

### 3.3 Three-dimensional collaborative intervention model

Based on a comprehensive analysis of literature evidence, policy guidance and practical experience, this study constructed a “three-dimensional six elements” (3D-6E) collaborative intervention theoretical model for patellar tendinopathy. The model construction followed a sequential integrative process: First, the systematic literature review identified evidence-based intervention components and their effectiveness parameters (e.g., progressive resistance training protocols, diagnostic criteria, assessment tools). Second, policy text analysis revealed the macro-level framework requirements and cross-sectoral collaboration mandates that the model must accommodate. Third, comparative case analysis identified operational mechanisms, facilitators, and barriers from real-world implementations. These three evidence streams were synthesized through an iterative process of framework development, where elements supported by convergent evidence across multiple sources were prioritized, and gaps identified in one source were addressed by findings from others. The resulting 3D-6E model thus represents an evidence-informed framework that bridges research findings, policy directives, and practical feasibility. This model expands from the traditional two-dimensional framework of physical education and medicine to the three-dimensional space of physical education, medicine and education, and systematically identifies the core construction elements of collaborative intervention. The results of the literature review show that progressive resistance training can increase the VISA-P score by 16.4 points (95% CI: 14.2–18.6), providing sufficient evidence support for the elements of exercise prescriptions. Policy analysis reveals that the number of provisions related to “health education” has increased from 1 in 2013 to 18 in 2024, a growth rate of 1,700%, highlighting the independent value of the education dimension. Case comparisons reveal the weak links in talent allocation and standardization of the existing model, providing practical basis for model optimization.

Before detailing the operational parameters of each element, it is important to summarize the convergent evidence supporting the model’s components. Progressive resistance training was consistently supported across the literature review (15 RCTs), policy documents (exercise prescription provisions increasing 1,700% from 2013 to 2024), and case studies (all four models incorporated exercise components). Standardized diagnostic protocols were endorsed by clinical guidelines and the Dutch multidisciplinary case. Patient education and empowerment received the strongest support from the policy evolution analysis, where health education provisions grew from 1 to 18 between 2013 and 2024, and was further validated by case evidence showing that models lacking structured education components exhibited higher recurrence rates. Exercise monitoring and evaluation feedback were supported primarily by clinical trial evidence and the Dutch guideline’s quality control framework. Early detection received moderate support, primarily from clinical guidelines and cohort study evidence regarding screening effectiveness.

The 3D-6E model proposed in this study contains three interrelated dimensions. The sports dimension covers two elements: Exercise Prescription and Exercise Monitoring. The exercise prescription is formulated based on the FITT principle, adopting a heavy-load slow resistance training program with a frequency of 3–4 times per week and an intensity of 6-8RM, combined with eccentric training and functional training. The intervention duration is a standard 12-week program. The training monitoring follows the principle of pain control, requiring the VAS score during training to be ≤3/10. The load management strategy is adopted to ensure the recovery of pain the next day. The advanced criterion is that the VISA-P score increases by ≥10 points every 4 weeks. The medical dimension integrates two elements: Evidence-based Diagnosis and Early Detection. A standardized process for evidence-based diagnosis was established, and a complete diagnostic pathway was formed through medical history collection, physical examination and imaging evaluation when necessary. Among them, the sensitivity of subpatellar extreme tenderness reached 85%, and the specificity of the Royal London Hospital Test reached 92%. Early detection focuses on screening and risk assessment of high-risk populations, and the clear referral indication is no improvement after 6 weeks of exercise therapy or an increase of VISA-*p* < 13 points after 12 weeks of treatment. The 13-point threshold for the minimal clinically important difference (MCID) of VISA-P was derived from the distribution-based method reported by Challoumas et al. (26), who established this value using anchor-based and distribution-based approaches in tendinopathy populations. It should be noted that the clinical significance of a 13-point improvement may vary depending on baseline severity: for patients with low baseline scores (e.g., VISA-P 20–30), achieving a 13-point improvement represents substantial functional gain, whereas for patients with higher baseline scores (e.g., VISA-P 60–70), the same absolute improvement may be proportionally less meaningful. Clinicians should therefore interpret MCID thresholds in conjunction with baseline severity and individual patient goals when making referral decisions. The educational dimension emphasizes the independent status of patient Empowerment and Evaluation feedback. Patient empowerment enhances disease awareness through a structured health education curriculum comprising four modules: (1) Understanding Patellar Tendinopathy (pathophysiology, healing timeline of 12–16 weeks, and prognosis); (2) Self-Monitoring Skills (maintaining a daily load monitoring diary using standardized templates, pain self-assessment using the Numeric Rating Scale); (3) Activity Modification Strategies (the 10% weekly load progression rule, recognition of warning signs); and (4) Relapse Prevention (identifying high-risk behaviors, gradual return-to-sport protocols). Educational delivery tools include printed patient handbooks, instructional videos, and mobile health applications that facilitate real-time symptom tracking and provide automated feedback. The evaluation feedback adopted a multi-dimensional index system, including VAS pain score, VISA-P function score (with a Minimal Clinically Important Difference (MCID) of 13 points), and SF-12 quality of life score, and was systematically evaluated at four time points: baseline, 6 weeks, 12 weeks, and 24 weeks. The feedback mechanism operates through a closed-loop process: assessment results are reviewed in multidisciplinary case conferences held biweekly, where clinicians, sports instructors, and health educators jointly determine whether to (a) continue the current protocol, (b) adjust exercise prescription intensity or frequency, (c) reinforce specific educational components, or (d) initiate specialist referral. Patients receive individualized progress reports at each assessment point, with visual representations of their improvement trajectory to enhance motivation and adherence.

The six elements form an organically integrated closed-loop system. Doctors identify patients’ conditions through evidence-based diagnosis. Qualified sports instructors—defined in this model as certified athletic trainers, licensed physiotherapists, or clinical exercise physiologists with formal training in musculoskeletal rehabilitation—develop personalized exercise prescriptions based on the diagnosis results. In the Chinese context, this role is typically fulfilled by professionals holding the National Social Sports Instructor Certificate (Level II or above) combined with additional training in exercise prescription, or by rehabilitation physicians and physical therapists licensed by the National Health Commission. Patients carry out training programs with the support of health education. Training monitoring ensures safety. Regular assessment and feedback adjust intervention strategies. Early detection mechanisms promptly identify cases that need to be referred. This collaborative mechanism breaks through the limitation of isolated operation of each professional field in the traditional model. From a systems perspective, the logic model framework presents the complete chain from investment to long-term impact. Policy support, financial guarantee, talent cultivation and facility construction are regarded as systematic investments. Through activities such as standardized diagnosis, exercise prescriptions, health education and effect evaluation, direct outputs such as the number of service recipients, the number of interventions and coverage are generated, and short-term results such as pain improvement, functional recovery and self-management ability enhancement are achieved. Ultimately, it leads to a long-term impact of reduced recurrence rates, increased participation in sports, reduced medical burdens, and improved overall health levels. The core elements of this model and their evidence-based basis are detailed in Table 3.

**Table 3:** Core elements and evidence base of the 3D-6E collaborative intervention model.

Dimension	Element	Core content	Key Parameters/Standards	Evidence level	References
Sport	Exercise Prescription (E1)	Progressive resistance training Eccentric + isometric + concentric combination	Frequency: 3–4 sessions/week Intensity: 6-8RM Time: 45–60 min/session Duration: 12 weeks	Low-Moderate (GRADE)	Ophey et al. (16) and Breda et al. (9)
	Exercise Monitoring (E2)	Pain monitoring Load management Progression criteria	VAS ≤ 3/10 to continue Pain recovery by next day VISA-P improvement ≥10 points/4 weeks	Moderate	Núñez-Martínez et al. (11)
Medicine	Evidence-based Diagnosis (E3)	Standardized diagnostic protocol Physical examination Imaging assessment	Inferior pole tenderness (sensitivity 85%) RLHT (specificity 92%) Consider after 12-week conservative failure	High	Ophey et al. (16) and Malliaras et al. (13)
	Early Detection (E4)	High-risk screening Risk assessment Referral criteria	BMI > 28, jumping athletes Load spike >10%/week No improvement at 6 weeks or VISA-P < 13 at 12 weeks	Moderate	Theodorou et al. (1)
Education	Empowerment (E5)	Disease awareness Self-management skills Relapse prevention	Healing timeline: 12–16 weeks Load monitoring diary, pain self-assessment Avoid sudden load increases	Moderate	Dinh and Bonner (21)
	Evaluation (E6)	Multi-dimensional indicators Assessment timepoints Feedback mechanism	VAS (0–10), VISA-P [0–100, MCID = 13 per Challoumas et al. (26); interpret with baseline consideration], SF-12 Baseline, 6, 12, 24 weeks Prescription adjustment, education reinforcement, referral decision	High	Nutarelli et al. (2)

E1-E6 represent Element 1–6; RLHT = Royal London Hospital Test; MCID = Minimal Clinically Important Difference; VAS = Visual Analogue Scale; VISA-P = Victorian Institute of Sport Assessment-Patella.

### 3.4 Comparative analysis of practice models

This study selects four representative practice models of the integration of sports and medicine for systematic comparative analysis, aiming to identify replicable successful experiences and systemic obstacles that need to be urgently addressed. The “Sunshine Fitness Card” model in Suzhou was launched in 2005. Through an economic incentive mechanism that binds medical insurance cards with fitness consumption, it has served over 500,000 residents in total, with an average annual participation rate growth of 12%, demonstrating the effectiveness of economic levers in promoting changes in public health behaviors. Since its implementation in 2016, the “1 + 1 + 1” community contract service model in Jiading District, Shanghai, has established a tripartite collaboration network involving community health service centers, district-level medical institutions and municipal hospitals. The coverage rate in the pilot areas has reached 85%. Through the family doctor contract system, it has achieved the first diagnosis at the grassroots level and hierarchical medical treatment, providing a feasible path for the integration of sports and medicine at the community level. The model of the National Physical Fitness Monitoring Station in Lucheng District, Wenzhou, combines physical fitness monitoring data with health guidance, establishing an integrated service process of “test - evaluation - guidance”. However, the depth of service still remains at the level of general health advice and lacks systematic intervention plans for specific diseases. As an international control case, the multidisciplinary guidelines of the Netherlands, with their diagnosis and treatment norms for patellar tendinopathy released in 2025, clearly define the collaboration interface between primary care and specialized care, have clear referral standards, and a complete quality control system, providing an important reference for Chinese practice.

Cross-case comparative analysis reveals three key success factors, as shown in Table 4. Government leadership is the common prerequisite for all successful models. Whether it is the innovation of medical insurance policies in the Suzhou model, the contracted service system in the Shanghai model, or the construction of the public service system in the Wenzhou model, they all rely on the policy support and financial investment of the government. The community, as the best implementation scenario, is particularly evident in the Shanghai model. Community health service centers, as collaborative hubs, have achieved the downward flow of medical resources and the accessibility of services. The effectiveness of economic incentives has been fully verified in the Suzhou model, and the expansion of the usage rights of medical insurance cards has directly enhanced the enthusiasm of residents to participate. However, the comparative analysis simultaneously exposed three types of systemic disorders. The bottleneck of talent is the most prominent. Social sports instructors generally lack medical knowledge, doctors have insufficient ability to prescribe exercise prescriptions, and there is a shortage of compound professionals with backgrounds in sports, medicine and education. The lack of standards seriously restricts the promotion of the model. 78% of the practical cases lack unified service process norms, the effect evaluation indicators vary, and the quality control mechanism is weak. The vulnerability of the coordination mechanism is reflected in the fact that cross-departmental collaboration remains at a superficial level, the sports, health and education departments operate independently, information systems have not been interconnected, and the unclear benefit distribution mechanism leads to insufficient collaborative motivation. The identification of these obstacles provides a clear direction for improvement in the formulation of subsequent optimization strategies.

**Table 4:** Comparison of sport-medicine integration practice models.

Model	Launch year	Coverage	Collaboration level	Standardization	Key strengths	Main barriers
Suzhou “Sunshine Fitness Card”	2005	>500,000 residents	★★☆ (Moderate)	χ (Absent)	Economic incentive effective; High participation	Lack of medical guidance; No disease-specific programs
Shanghai Jiading “1 + 1 + 1”	2016	85% in pilot areas	★★★ (High)	★★ (Partial)	Strong primary care network; Clear referral pathways	Limited sports integration; Insufficient exercise prescription capacity
Wenzhou Lucheng Physical Fitness Monitoring	2012	30,000 tests/year	★☆ (Low)	★ (Basic)	Systematic fitness assessment; Public facility utilization	Shallow service depth; No medical collaboration
Dutch Multidisciplinary Guideline	2025	National coverage	★★★★ (Very High)	★★★★ (Comprehensive)	Clear diagnostic criteria; Defined referral thresholds; Quality assurance	Resource-intensive; Requires specialized training

Collaboration Level: ★☆ = Minimal; ★★☆ = Moderate; ★★★ = High; ★★★★ = Very High. Standardization: χ = Absent; ★ = Basic; ★★ = Partial; ★★★ = Substantial; ★★★★ = Comprehensive.

## 4 Discussion

This study conducted a modeling and path-based exploration on the collaborative intervention of physical, medical and educational approaches for patellar tendinopathy. The results are generally consistent with the evidence-based evidence in recent years and provide operational Chinese contextualized supplements in several aspects. Existing high-quality reviews and guidelines suggest that the hierarchical management centered on exercise therapy remains the first-line solution, but the collaborative chain at the implementation level, educational empowerment, and quality control are still weak links (16). Compared with previous literature that mainly focused on the effectiveness of a single intervention, this study takes the collaboration of “multiple departments - multiple specialties - multiple scenarios” as the basic assumption in the path design, and responds to the question of “how to implement” with process standards and evaluation closed-loop. It is important to clarify the relative strength of evidence supporting each component: exercise prescription and progressive loading protocols are most strongly supported by high-quality RCT evidence; evidence-based diagnosis benefits from well-established clinical guideline consensus; patient empowerment and educational components, while strongly supported by policy evolution data and behavioral theory, currently rely primarily on observational and secondary sources rather than direct experimental evidence in tendinopathy populations; and the effectiveness of the integrated multidisciplinary collaboration model as a whole derives mainly from observational comparisons, with the relative contribution of each individual component yet to be determined through dismantling studies or factorial designs. This approach is in line with the recent umbrella review and clinical management review on the evidence map of patellar tendinopathy (22, 23). In terms of specific prescriptions and training strategies, this study advocates for prescription stratification based on a combination of progressive loading, centrifugation/isometric loading, and Heavy Slow Resistance (HSR), and sets on-site assessment and referral thresholds. This is consistent with the comparison conclusions of different exercise programs in online reviews, while also taking into account the key points of load management from the perspectives of season and prevention (27, 28). For the evidence of soft tissue manipulation combined with exercise training, the effect size and certainty of different research reports vary. This study positions it as an optional complementary measure and reduces the risk of variation through a monitoring-feedback mechanism (29).

In terms of comprehensive rehabilitation strategies, this study acknowledges the complexity of treatment options. Network meta-analysis has examined multiple injection strategies, though the relative effects remain uncertain and require further investigation (30); Evidence from randomized controlled trials suggests that platelet-rich plasma may offer context-dependent benefits for symptom improvement (31); Additionally, comparative studies have shown that different modalities such as focused and radial shockwave therapy demonstrate similar effectiveness, suggesting device selection should be individualized (32).

Compared with existing studies, the main contribution of this study lies in extrapolating the consensus requirements of the military and sports population (emphasizing task and return standards) into operable collaborative standards for the general population, and detailing them to cross-departmental responsibility division and information sharing interfaces, enhancing the feasibility of transformation and promotion (33); Meanwhile, an evaluation framework oriented by MCID based on scales such as VISA-P was introduced to make the interpretation of therapeutic effects more clinically significant (26). However, this study also has some shortcomings: First, the evidence integration still mainly comes from secondary research and extrapolation from international experience, lacking multi-center prospective validation (34); Secondly, the proposed collaborative process and quality indicators have not yet been systematically evaluated by health economics and implementation science (35). Thirdly, regional resource differences, the heterogeneity of grassroots talent structure, and payment policies may affect the replicability and sustainability of the model. Specifically, regions with well-established sports medicine infrastructure and higher densities of qualified exercise professionals (e.g., eastern coastal provinces) may achieve more rapid and complete model implementation, whereas resource-limited regions with fewer trained professionals and less developed cross-departmental coordination mechanisms may require simplified or phased implementation approaches. The case comparison findings illustrate this variability: Suzhou and Shanghai models benefited from strong municipal government support and established healthcare networks, whereas the Wenzhou model’s limited service depth reflects the constraints of less developed cross-sectoral infrastructure. Future implementation strategies should therefore incorporate region-specific adaptation protocols that account for local talent availability, funding mechanisms, and existing policy frameworks. Future research should conduct embedded implementation experiments, combined with hierarchical randomization or cluster design, to verify the effectiveness and cost-effectiveness of medical education collaboration at different levels and among different populations. Based on real-world data, a continuous improvement system driven by both outcome and service quality dimensions should be established to gradually perfect the evidence closed loop and policy closed loop.

## 5 Conclusion

This study constructed a preliminary “sport-medicine-education” three-dimensional collaborative (3D-6E) intervention framework for patellar tendinopathy, confirming that exercise therapy with progressive loading as the core, combined with standardized assessment and health education, can improve function and compliance in process-oriented management. Adopt a cautious attitude towards second-line methods such as injection and physical factors, and emphasize evidence-based thresholds and the closed loop of re-evaluation. The model is operational in terms of cross-departmental responsibilities, information sharing and quality control, but it still requires multi-center prospective validation and health economics assessment. The research provides path references for policy implementation and service system optimization, and lays the foundation for subsequent real-world research.

## Data Availability

The original contributions presented in the study are included in the article/Supplementary material, further inquiries can be directed to the corresponding author/s.
