# Supplementary material for: Construction elements and preliminary framework of a sport-medicine-education collaborative intervention model for patellar tendinopathy
Source: Front Public Health. 2026 Mar 17;14:1729240. doi: 10.3389/fpubh.2026.1729240 (PMC13036120; doi:10.3389/fpubh.2026.1729240)
Supplement: Supplementary file 2 [file Table_1.docx]

**Supplementary Table S1. Complete Search Strategies for All Databases**

| **Database** | **Search Block** | **Search Terms** |
| --- | --- | --- |
| **PubMed** | #1 Disease terms | "Patellar Tendinopathy"[MeSH Terms] OR "patellar tendinopathy"[Title/Abstract] OR "jumper's knee"[Title/Abstract] OR "patellar tendinosis"[Title/Abstract] OR "patellar tendon disease"[Title/Abstract] |
|  | #2 Intervention terms | "Exercise Therapy"[MeSH Terms] OR "Rehabilitation"[MeSH Terms] OR "exercise therapy"[Title/Abstract] OR "rehabilitation"[Title/Abstract] OR "resistance training"[Title/Abstract] OR "eccentric exercise"[Title/Abstract] OR "physical therapy"[Title/Abstract] |
|  | #3 Collaborative model terms | "Integrative Medicine"[MeSH Terms] OR "Patient Care Team"[MeSH Terms] OR "integrative medicine"[Title/Abstract] OR "collaborative care"[Title/Abstract] OR "multidisciplinary"[Title/Abstract] OR "interdisciplinary"[Title/Abstract] |
|  | #4 Combined | #1 AND #2 AND #3 |
|  | Filters | Publication date: 2013/01/01 to 2024/10/31; Languages: English, Chinese |
| **Web of Science** | #1 Disease terms | TS=("patellar tendinopathy" OR "jumper's knee" OR "patellar tendinosis" OR "patellar tendon disease") |
|  | #2 Intervention terms | TS=("exercise therapy" OR "rehabilitation" OR "resistance training" OR "eccentric exercise" OR "physical therapy" OR "exercise intervention") |
|  | #3 Collaborative model terms | TS=("integrative medicine" OR "collaborative care" OR "multidisciplinary" OR "interdisciplinary" OR "patient education") |
|  | #4 Combined | #1 AND #2 AND #3 |
|  | Filters | Timespan: 2013-2024; Languages: English, Chinese |
| **Scopus** | #1 Disease terms | TITLE-ABS-KEY("patellar tendinopathy" OR "jumper's knee" OR "patellar tendinosis" OR "patellar tendon disease") |
|  | #2 Intervention terms | TITLE-ABS-KEY("exercise therapy" OR "rehabilitation" OR "resistance training" OR "eccentric exercise" OR "physical therapy") |
|  | #3 Collaborative model terms | TITLE-ABS-KEY("integrative medicine" OR "collaborative care" OR "multidisciplinary" OR "interdisciplinary") |
|  | #4 Combined | #1 AND #2 AND #3 |
|  | Filters | PUBYEAR > 2012 AND PUBYEAR < 2025; LANGUAGE(English OR Chinese) |
| **Cochrane Library** | #1 Disease terms | MeSH descriptor: [Tendinopathy] explode all trees OR "patellar tendinopathy":ti,ab,kw OR "jumper's knee":ti,ab,kw OR "patellar tendinosis":ti,ab,kw |
|  | #2 Intervention terms | MeSH descriptor: [Exercise Therapy] explode all trees OR MeSH descriptor: [Rehabilitation] explode all trees OR "exercise therapy":ti,ab,kw OR "rehabilitation":ti,ab,kw |
|  | #3 Collaborative model terms | MeSH descriptor: [Integrative Medicine] explode all trees OR "collaborative care":ti,ab,kw OR "multidisciplinary":ti,ab,kw |
|  | #4 Combined | #1 AND #2 AND #3 |
|  | Filters | Publication Year from 2013 to 2024 |
| **SPORTDiscus** | #1 Disease terms | TI("patellar tendinopathy" OR "jumper's knee" OR "patellar tendinosis") OR AB("patellar tendinopathy" OR "jumper's knee" OR "patellar tendon disease") |
|  | #2 Intervention terms | TI("exercise therapy" OR "rehabilitation" OR "resistance training") OR AB("exercise therapy" OR "rehabilitation" OR "eccentric exercise" OR "physical therapy") |
|  | #3 Collaborative model terms | TI("integrative medicine" OR "collaborative care" OR "multidisciplinary") OR AB("integrative medicine" OR "collaborative care" OR "interdisciplinary") |
|  | #4 Combined | S1 AND S2 AND S3 |
|  | Filters | Published Date: 20130101-20241031; Language: English, Chinese |
| **CNKI (中国知网)** | #1 疾病相关词 | SU='髌腱病' OR SU='髌腱末端病' OR SU='跳跃膝' OR SU='髌腱炎' |
|  | #2 干预措施词 | SU='运动疗法' OR SU='康复' OR SU='抗阻训练' OR SU='离心训练' OR SU='物理治疗' OR SU='运动处方' |
|  | #3 协作模式词 | SU='体医融合' OR SU='多学科协作' OR SU='协同护理' OR SU='健康教育' OR SU='跨学科' |
|  | #4 Combined | (#1) AND (#2) AND (#3) |
|  | 限定条件 | 发表时间：2013-2024；来源类别：学术期刊 |
| **Wanfang Data (万方数据)** | #1 疾病相关词 | 主题:(髌腱病 OR 髌腱末端病 OR 跳跃膝 OR 髌腱炎) |
|  | #2 干预措施词 | 主题:(运动疗法 OR 康复 OR 抗阻训练 OR 离心训练 OR 物理治疗) |
|  | #3 协作模式词 | 主题:(体医融合 OR 多学科协作 OR 协同护理 OR 健康教育) |
|  | #4 Combined | #1 AND #2 AND #3 |
|  | 限定条件 | 年份：2013-2024；文献类型：期刊论文 |
| **VIP (维普数据库)** | #1 疾病相关词 | M=(髌腱病+髌腱末端病+跳跃膝+髌腱炎) |
|  | #2 干预措施词 | M=(运动疗法+康复+抗阻训练+离心训练+物理治疗) |
|  | #3 协作模式词 | M=(体医融合+多学科协作+协同护理+健康教育) |
|  | #4 Combined | #1 AND #2 AND #3 |
|  | 限定条件 | 时间范围：2013-2024 |
| **CBM (中国生物医学文献数据库)** | #1 疾病相关词 | ("髌腱病"[常用字段:智能] OR "髌腱末端病"[常用字段:智能] OR "跳跃膝"[常用字段:智能] OR "髌腱炎"[常用字段:智能]) |
|  | #2 干预措施词 | ("运动疗法"[常用字段:智能] OR "康复"[常用字段:智能] OR "抗阻训练"[常用字段:智能] OR "物理治疗"[常用字段:智能]) |
|  | #3 协作模式词 | ("体医融合"[常用字段:智能] OR "多学科协作"[常用字段:智能] OR "健康教育"[常用字段:智能]) |
|  | #4 Combined | #1 AND #2 AND #3 |
|  | 限定条件 | 发表年份：2013-2024 |

***Abbreviations:*** *MeSH = Medical Subject Headings; TI = Title; AB = Abstract; TS = Topic; SU = Subject; M = Theme.*

***Note:*** *The search was conducted from January 2013 to October 2024. Language restrictions were applied to include only English and Chinese publications. The search strategy combined disease-related terms (patellar tendinopathy, jumper's knee), intervention terms (exercise therapy, rehabilitation), and collaborative model terms (integrative medicine, collaborative care) using Boolean operators, as described in the Methods section.*

**Supplementary Table S2. Risk of Bias Assessment Results for Included Studies**

| **Study Type / Assessment Tool** | **Assessment Domain** | **Result** | **Main Limitations** |
| --- | --- | --- | --- |
| **RCTs (n=15)** | Random sequence generation | Low risk: 8 (53.3%) | — |
| Cochrane RoB 2.0 | Allocation concealment | Low risk: 6 (40.0%) | — |
|  | Blinding of outcome assessment | Low risk: 4 (26.7%) | Difficult to blind |
|  | Incomplete outcome data | Low risk: 10 (66.7%) | — |
|  | Selective reporting | Low risk: 12 (80.0%) | — |
| **Systematic Reviews (n=8)** | High quality | 5 (62.5%) | — |
| AMSTAR-2 | Moderate quality | 2 (25.0%) | Appraisal reporting |
|  | Low quality | 1 (12.5%) | No protocol registration |
| **Cohort Studies (n=6)** | Selection (max 4 stars) | Mean: 3.2 stars | — |
| Newcastle-Ottawa Scale | Comparability (max 2 stars) | Mean: 1.3 stars | Confounding control |
|  | Outcome (max 3 stars) | Mean: 2.7 stars | — |
|  | Overall (max 9 stars) | Mean: 7.2 stars | — |
| **Clinical Guidelines (n=3)** | Scope and purpose | Mean: 85.2% | — |
| AGREE II | Rigor of development | Mean: 78.3% | — |
|  | Clarity of presentation | Mean: 88.7% | — |
|  | Applicability | Mean: 52.8% | Weakest domain |
|  | Overall (7 domains) | Mean: 5.8/7 (76.5%) | — |

***Note:*** *For RCTs, blinding of participants and outcome assessors was the main source of bias due to the inherent difficulty of blinding exercise interventions. For systematic reviews, lack of protocol registration was the primary limitation. For cohort studies, inadequate control for confounding variables was the most common weakness. For clinical guidelines, applicability was consistently the weakest domain.*
